# The State Medical Society of New York and the New Code

**Published:** 1883-03

**Authors:** 


### THE STATE MEDICAL SOCIETY OF NEW YORK AND THE NEW CODE.

At the late meeting of the New York State Medical Society it was resolved by a vote of 105 to 99 to stand by the action of last year regarding the code and to decline sending delegates to the American Medical Association.

This action of the society was a surprise to almost everyone, for it was generally believed that the overwhelming sentiment of the profession of the State was opposed to this scheme of the city specialists, and that the loyal element would so express itself in opposition to the innovation as to secure the rescinding of the obnoxious substitute for the old code adopted at the preceding session.

This deliberate action of the society simply cuts it off from affiliation with the American Medical Association and relieves that body from the unpleasant duty of dealing with the question in a much more decisive manner than it did last year. The effort to coerce the Association into measures agreeable to the pockets of a few specialists in New York city has failed, and it now only remains for the more disinterested, conservative and wiser portion of the society to save themselves from embarrassment and to protect their reputations by promptly withdrawing from a body which fails utterly to appreciate the spirit, and which now seeks to set aside the letter of the code.

### ANNOUNCEMENT.

In consequence of certain contemplated business changes on the part of the proprietor of the REGISTER, and of the exacting demands of other important duties upon the time and attention of the editor, the agreement heretofore existing between the publisher and the editor has been canceled by mutual consent, and with the next number of this journal the present editorial management will cease.

